# Penile Mondor’s disease after anterolateral retroperitoneal approach for lumbar fracture

**DOI:** 10.1136/bcr-2017-220790

**Published:** 2017-11-01

**Authors:** Mauro Dobran, Roberta Benigni, Davide Nasi, Daniele Cantoro

**Affiliations:** 1 Neurosurgery, Neurosurgery, Ancona, Italy, Italy; 2 Urology, Ancona, Italy

**Keywords:** venous thromboembolism, neurological injury, neurosurgery, urological surgery

## Abstract

This is a rare case of thrombosis of the dorsal vein of the penis (Mondor’s disease) occurred after an anterior-lateral retroperitoneal approach for a vertebral stabilisation in thoracolumbar vertebral fracture. Potential causes are traumatism, neoplasms, excessive sexual activity or abstinence. Although penile Mondor’s disease is a clinical diagnosis, ultrasound imaging is the gold standard to confirm it. In the reported case, 1 week after neurosurgical retroperitoneal procedure of vertebral stabilisation, the patient complained of a painful cord-like mass midshaft of penis. The diagnosis was made by clinical evaluation and ultrasound images. After 2 weeks of therapy with enoxaparin sodium, the patient recovered. The authors report this case evaluating the possible correlation between the anterior-lateral retroperitoneal approach and the development of the rare Mondor’s disease.

## Background

Mondor’s disease is a thrombophlebitis of the superficial veins of the body and it was described by Enri Mondor first time in 1939.^1^ Thrombosis of the superficial dorsal vein of the penis was described by Braun Falco in a patient with a generalised phlebitis in 1958. Isolated thrombosis of the dorsal superficial vein of penis was first reported by Helm and Hodge in 1958.^2^ Still today, a few cases of isolated thrombophlebitis of the superficial vein of penis are reported in literature.^3^ No specific aetiology has been found, and patients’ age ranged between 18 and 79 years. Hypothetical causes of disease include trauma, infections, excessive sexual activity or abstinence, thrombophilia, inguinal hernia, body building exercises, pelvic cancer, pelvic venous occlusion and stasis. In the described case, the spinal stabilisation was performed with left subcostal retroperitoneal approach. During surgery, excessive compression of venous structures by self-retractors and coagulation of abdominal veins were avoided. In this patient, a prompt diagnosis allowed to treat the rare disease and prevent serious complications such as vein occlusion with consequent resection of the dorsal vein of the penis.

## Case presentation

**Figure 1:**
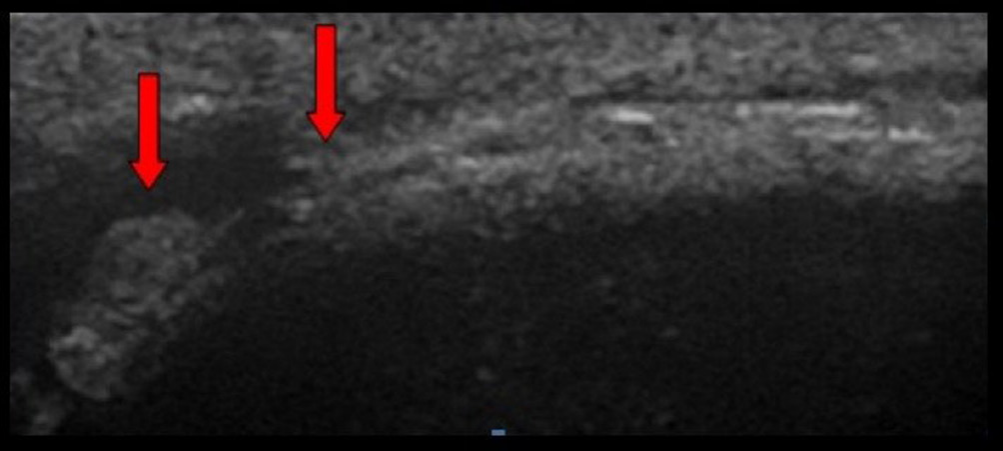
Ultrasound image showing thrombosis of the superficial dorsal vein of the penis (arrows).

**Figure 2:**
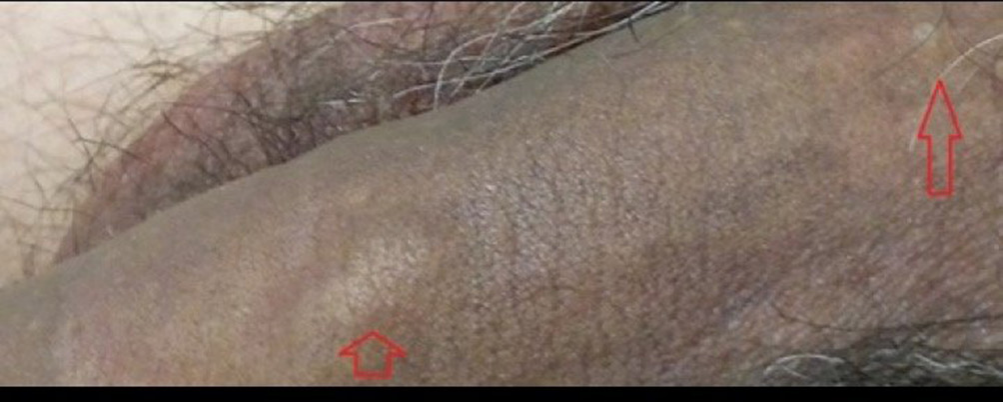
Arrows indicated the superficial dorsal vein thrombosis.

A 31-year-old man, smoker of 30 cigarettes a day, was admitted to our department with a vertebral trauma and a burst fracture of L1 without neurological deficit. His body mass index was 25. The first procedure was a posterior stabilisation D12-L2 with rods and screws. One month later, a second surgical procedure was performed by an anterior-lateral retroperitoneal left side approach. The patient was positioned in lateral right position with flexion of the legs. A subcostal incision was made, and by a retroperitoneal approach, screws T12-L2 were implanted in one plate. Six-hour surgery was conducted, and no vascular injury was reported. Three days later, the patient walked with a stick. In accordance with our standard protocol, mechanical deep venous prophylaxis and chemoprophylaxis (enoxaparine sodium 4000 UI/day) started the day of surgery and went on for 3 days. After 7 days, at discharge, the patient complained of pain and dorsal induration of the penis. The pain was throbbing. No fever, dysuria and no typical signs of inflammation were present in the serum. Patient clinical examination documented palpable thick cord-like lesion on the dorsal side of his penis, the skin was absolutely intact with no redness (figure 1). There were no signs of lymphadenopathy in the groin region. Ultrasound images revealed a segmental thrombosis of the superficial dorsal vein of the penis (figure 2), which appeared as internal echogenicity without flow signal at colour Doppler sonography.^4^ Haemocoagulative screening was negative for thrombophilia. Abdominal CT scan did not show venous thrombosis in abdominal veins. Therapy started with enoxaparine sodium 4000 UI/day and antibiotics (levofloxacin 500 mg twice a day for a week). The patient was reassured about the benign nature of his disease and was discharged 2 days later.

## Outcome and follow-up

Presenting clinical symptoms and signs resolved after 2 weeks of therapy and ultrasound images documented the complete reopening of the vein. At 3-month follow-up examination, there were no findings of disease (both clinical and ecographic), the patient referred no sexual dysfunctions and enoxaparine was stopped.

## Discussion

The thrombosis of the dorsal vein of the penis is a rare condition with only 49 articles found in the literature.^5^ Correct and fast diagnosis grants a prompt recovery. Venous drainage of penis starts at the base of the glans, then the circumflex veins extend around the corpus cavernosum and reach the dorsal vein perpendicularly. This network of veins reaches the pudendal veins flowing into internal iliac vein that join the external iliac vein to form the common iliac vein that reach the vena cava. In polytraumatised patients with plurifragmental vertebral fractures, the posterior approach with screws and rods may be insufficient to grant stability^6 7^ especially in the short fixation. In these patients or in case of removal of the posterior instrumentation for infection,^8^ a second anterior-lateral approach and stabilisation with vertebral plate is mandatory. In the present case, the surgical approach was retroperitoneal on the left side with the patient in lateral position on the right side with flexion of the legs. The mean surgical procedure time was 6 hours in most of the cases. To prevent complications and stasis, the correct positioning of the patient on the operating table is imperative. Postsurgical vascular stasis has been suggested as a cause of the thrombosis^9–11^ due to prolonged immobility.^12 13^ A potential complication of anterior vertebral surgery is the vascular injury of the left common iliac vein.^14^ It is important to release the traction of handheld retractors at regular intervals no longer than 15 min to avoid compression and thrombosis.^15^ In the described case, we assume that the prolonged position on the patient’s right side with flexion of the legs may have caused a pelvic venous stasis with subsequent thrombosis of the dorsal penile vein. Another potential cause may have been the crushing of the penis on the operating table and finally the visceral compression by the self-retaining retractor during the long surgical procedure. Another potential cause may be the tendency to thrombosis of the patient (no documented by haematological screening) who 6 months before the vertebral surgery presented a deep vein thrombosis in the leg without permanent sequel or recurrence. Anyway it is important to diagnose the disease by means of ultrasound images and make differential diagnosis from the non-venereal sclerosing lymphangitis. Our patient recovered with enoxaparin sodium while other patients got spontaneous resolution without therapy.^3^ There is some evidence that people affected with Mondor’s disease are more likely to relapse if predisposing factors persist.^16^ Finally, most cases resolve within 4 to 6 weeks, in persistent cases surgery with a thrombectomy or resection of the superficial dorsal vein may be necessary.^17^ Thrombosis of the superficial vein of the penis is a rare benign disease. No specific aetiology has been found but the pelvic venous stasis may be a cause in operated patients. It is important to diagnosis the disease as soon as possible to start an adequate therapy. The diagnosis is made by clinical evaluation and ultrasound imaging.

**Learning points**

- Mondor’s disease is a benign thrombophlebitis of superficial veins.
- The thrombosis of the superficial dorsal veins of the penis is a rare benign complication of pelvis stasis.
- In spinal surgery with anterior-lateral position, it is important to prevent veins compression or the crushing of the penis on the operating table.
- Ultrasound images are the gold standard for the diagnosis.
